# Investigating Factors Associated with Depressive Symptoms of Chronic Kidney Diseases in China with Type 2 Diabetes

**DOI:** 10.1155/2017/1769897

**Published:** 2017-02-02

**Authors:** Xu Wang, Biyu Shen, Xun Zhuang, Xueqin Wang, Weiqun Weng

**Affiliations:** ^1^Department of Nursing, The Second Affiliated Hospital of Nantong University, Nantong, Jiangsu Province, China; ^2^School of Nursing, Nantong University, Nantong, Jiangsu Province, China; ^3^Department of Epidemiology, School of Public Health, Nantong University, Nantong, Jiangsu Province, China; ^4^Department of Endocrinology, The Second Affiliated Hospital of Nantong University, Nantong, Jiangsu Province, China

## Abstract

*Aim.* To assess the depressive symptoms status of chronic kidney diseases in Nantong, China, with type 2 diabetes and to identify factors associated with depressive symptoms.* Methods.* In this cross-sectional analytic study, 210 type 2 diabetic patients were recruited from the Second Affiliated Hospital of Nantong University. Depressive symptoms were assessed with the depression subscale of the Hospital Anxiety and Depression Scale (HAD-D). The quality of life was measured with the RAND 36-Item Health Survey (SF-36). And the independent risk factors of depressive symptoms were assessed by using a stepwise forward model of logistic regression analysis.* Results.* The mean age of the study subjects was 57.66 years (SD: 11.68). Approximately 21.4% of subjects reported depressive symptoms (*n* = 45). Forward stepwise logistic regression analysis showed that female gender (*P* = 0.010), hypertension (*P* = 0.022), Stage IV (*P* = 0.003), and Stage V (*P* < 0.001) were significant risk factors for depressive symptoms. The quality of life of individuals with HAD-D score <11 was significantly better compared with individuals with HAD-D score ≥ 11.* Conclusions.* These results indicate that clinicians should be aware that female patients with chronic kidney diseases with T2DM in their late stage with hypertension are at a marked increased risk of depressive symptoms. Providing optimal care for the psychological health of this population is vital.

## 1. Introduction

In China, high rates of unhealthy diets, physical inactivity, and obesity have led to an increase in the prevalence of type 2 diabetes mellitus (T2DM). Over 100 million Chinese people currently suffer from T2DM. T2DM is a chronic disease that in the long-term increases the microvascular degenerative complication of chronic kidney disease (CKD), which is a leading cause of end-stage renal disease (ESRD) [1]. ESRD requires either dialysis or transplantation, which is associated with cumulative hospital days and number of hospitalizations, contributing to excessive Medicare costs [2, 3]. CKD affects 20% to 40% of those who develop diabetes [4]. As the prevalence of T2DM in this country is increasing, the rate of CKD will also rise rapidly.

Depression is the third most important cause of disability in the world [5]. And having T2DM increases the risk of subsequent development or recurrence of depression. As an unwanted cotraveler of T2DM, depression can impinge on self-management ability and hinder patients adherence to treatment regime [6]. Furthermore, available studies have suggested that the presence of comorbid depression with diabetes is associated with poorer self-reported health status and higher adverse outcomes, including incident ESRD and mortality [7–13].

According to other documents, the prevalence of depression is observed in approximately 20% of patients with T2DM [14, 15]. Considering the high prevalence of CKD in patients with diabetes and that depression is rather common in patients with T2DM, the factors associated with depression effect on CKD in T2DM are a growing concern.

To our knowledge, the associated factors with depression were younger age, female gender, socioeconomic status, presence of cardiovascular disease, and health status [16, 17]. Furthermore, depression is associated with microalbuminuria in diabetes related CKD [18]. And low eGFR was regarded as a risk factor of depression in nondiabetic CKD [19, 20].

However, the factors associated with depressive symptoms among CKD in type 2 diabetic patients have not been examined in previous studies. Therefore we were specifically interested in the relationship between demographic, clinical variables, and depressive symptoms in CKD patients with T2DM.

The aim of this study was to assess the associations between depressive symptoms and kidney disease in an inpatient type 2 diabetic cohort and to learn about the warning signs of mood symptoms in these patients to provide early diagnosis and treatment.

## 2. Methods

### 2.1. Study Population and Recruitment

From October 2015 to April 2016, an observational cross-sectional study was conducted among the T2DM patients with CKD from the Second Affiliated Hospital of Nantong University. An eligible person fulfilled the 1999 World Health Organization (WHO) criteria for the diagnostic of T2DM or a hospital discharge diagnosis of T2DM. When abnormal estimated glomerular filtration rate (eGFR) or urinary albumin excretion rate (UAER) was presented in three months, T2DM patients would be categorized as having CKD. The severity of CKD in T2DM was categorized into five stages according to NKF-KDOQI Guidelines [21, 22] and the staging standard of CKD in type 1 diabetes [23]. Stage I: UAER < 10 *μ*g/min and eGFR increased obviously, kidney size increased; Stage II: UAER < 20 *μ*g/min or increased intermittently (such as exercise, stress state), eGFR increased slightly; Stage III: UAER 20~200 *μ*g/min (last for 3 months), eGFR is normal or above normal; Stage IV: UAER > 200 *μ*g/min, eGFR decreased; Stage V: end-stage renal failure, eGFR < 10 ml/min. eGFR was estimated from modified MDRD equations—c-aGFR4 [24–26]. Cases were excluded on the basis of the following criteria: (1) age < 18 or >80 years at the time of interview; (2) pregnant and lactating women; (3) hearing and cognitive impairment; (4) any unstable medical illness or mental disease; and (5) those who did not complete the questionnaire. Finally, 210 patients were eligible for investigation and entered the study.

In compliance with the Helsinki declaration, all subjects were told about the concept of the study and signed an informed consent prior to commencement of the study. Approval to conduct this study was obtained from the Ethics Committee of the Second Affiliated Hospital of Nantong University.

### 2.2. Instruments

Two scales, the Hospital Anxiety and Depression Scale (HAD) and the RAND 36-Item Health Survey (SF-36), were used to evaluate depressive symptoms and quality of life (QOL). The HAD [27], a reliable and valid self-report rating scale [28], includes 7 items for anxiety (HAD-A) and 7 for depression (HAD-D). The total score is the sum of the 14 items with a four-point scale on each item (range 0–3). For each subscale, the score is the sum of the respective seven items (ranging from 0 to 21). We defined the scores of HAD-D greater than 11 as depressive symptoms.

QOL were assessed with the SF-36, comprising 36 items organized into 8 scales: physical functioning (PF) (10 items), role physical (RP) (4 items), body pain (BP) (2 items), general health (GH) (5 items), energy/fatigue (VT) (4 items), social functioning (SF) (2 items), role emotional (RE) (3 items), and mental health (MH) (5 items). In addition to the above 8 aspects, SF-36 also contains another health indicator: reported health transition (HT) (1 item), used to evaluate the health status of the overall change in the past year. A Chinese version of the SF-36 scale has been validated by Wang et al. [29].

Each participant underwent an in-person interview of demographic data (age, gender, income, level of education, health coverage, etc.), diabetes characteristics (duration of diabetes, insulin therapy), and self-care behavior (clinic visit, blood glucose monitoring). Detailed clinical parameters (hypertension, HbA1c, plasma creatinine, and albuminuria) were abstracted from electronic medical record system.

### 2.3. Statistical Analysis

Statistical analyses were performed with SSPS 21.0. Descriptive statistics were calculated for all variables measured. Data were reported as mean (standard deviation [SD]) or median (interquartile range, IQR) or percentages. Significant differences were determined by using independent *t*-tests (continuous variables with normal distribution), Chi-Square tests (categorical variables), and Mann–Whitney tests (continuous variables with skewed distribution). A stepwise forward model of logistic regression was used to determine independent factors of depressive symptoms with odds ratios (ORs) and the corresponding 95% CIs. Statistical significance was set at *P* < 0.05 (two tailed).

## 3. Results

A total of 223 patients were invited to participate. Thirteen (5.8%) were excluded because of incomplete data. Among the 210 total subjects, 45 (21.4%) patients with T2DM had symptoms of depression. In Tables 1(a) and 1(b), for HAD-D scores ≥ 11 patients, significant associations were found with female gender (*P* = 0.006), yearly income (*P* = 0.016), clinic visit frequency (*P* = 0.018), blood glucose monitoring frequency (*P* = 0.019), and duration of diabetes (*P* = 0.035). The HAD-D scores ≥ 11 were higher in T2DM complicated with hypertension (27.8%) compared with T2DM without hypertension (13.7%) (*χ*^2^ = 6.18; *P* = 0.013). The rates of depressive symptoms among patients in Stage IV and V were higher than patients in I–III (*χ*^2^ = 23.28; *P* < 0.001). About forty percent of the patients who reported depressive symptoms were found with low eGFR (*χ*^2^ = 15.42; *P* < 0.001) and macroalbuminuria (*χ*^2^ = 17.49; *P* < 0.001). However, there were no significant differences in depressive symptoms by age (*P* = 0.533), employment status (*P* = 0.290), education (*P* = 0.150), marital status (*P* = 0.093), insurance (*P* = 0.117), insulin use (*P* = 0.334), or HbA1c (*P* = 0.921).

Forward stepwise logistic regression analysis was used to identify a model to predict T2DM-related CKD patient who would have depressive symptoms. The results showed that variables of female gender, severity of T2DM-related CKD, and complicated hypertension were significant risk factors for depressive symptoms (Table 2). This model had a good fit under the Hosmer-Lemeshow goodness-of-fit test (*R*^2^ = 0.233, *χ*^2^ = 7.568, *P* = 0.477), and accuracy of the model was 78.5%.

T2DM patients with hypertension were 2.5 times more likely to experience depressive symptoms than T2DM patients without hypertension (*P* = 0.022). The likelihood of depressive symptoms in Stage IV was 6.5 times more than that in stage I, and Stage V was 11.8 times. Female patients were 2.6 times more likely to report depressive symptoms than male patients.

Finally, the subjects were divided into two groups according to the HAD-D score. An analysis showed that all the dimension scores of SF-36 in individuals with HAD-D score < 11 were significantly higher compared with individuals with HAD-D score ≥ 11 (*P* < 0.05). The results are shown in Table 3.

## 4. Discussion

We found that almost 21.4% of subjects had depressive symptoms (HAD-D score ≥ 11). And the depressive symptoms were negatively associated with patients' quality of life. Depressive symptoms are becoming a serious problem.

We also found the differences between T2DM patients with and without depressive symptoms were yearly income, clinic visit frequency, blood glucose testing frequency, diabetic duration, eGFR, and albuminuria by univariate analysis. The lower compliance to the self-care measures of clinic visit and blood glucose testing will lead to a poor glycemic control which can contribute to the occurrence of diabetic complications [30, 31]. Besides, an increased duration of T2DM contributes significantly to an increase in the risk of diabetic complications. Low eGFR and macroalbuminuria revealed severe renal impairment which could require dialysis or kidney transplantation. These adverse consequences will lead to the increased economic burden of healthcare costs for lower income group. Due to economic instability and increased healthcare expenditures for complications and adherence to treatment, these patients are more susceptible to experiencing psychological diseases [32].

However, age, being unmarried, low monthly income, low educational level, unemployment, and HbA1c associated with depressive symptoms occurrence in T2DM patients have been proved in previous studies [30, 33–36]. These associations were not revealed in our 210 patients. We expect that this is due to a relatively low power, caused by the relatively low number of cases experiencing depressive symptoms. This is a main limitation of our study. Besides, variations in study design and participants' demographic characteristics might also be the reasons for the discrepancy.

In the final stepwise logistic regression we found that female gender was an independent risk factor for depressive symptoms, which was in line with previous studies [37, 38]. Sun et al. [39] found an association between being woman and depressive symptoms. This sex difference could be due to lower physical activity lifestyle and being more emotional than men.

Besides being female, hypertension was also an independent risk factor for depressive symptoms among T2DM-related CKD patients by stepwise logistic regression. Based on the previous studies [40, 41], hypertension has been demonstrated to be an independent risk factor of depressive symptoms among patients with T2DM due to increased risk of serious cardiovascular disease complications, reduced quality of life, and poor prognosis.

Additionally, the stepwise logistic regression analysis also indicated that the T2DM-related CKD progression was significantly associated with depressive symptoms. CKD with type 2 diabetic patients were reported to experience symptoms like inability to sleep, depression, and lack of energy when they perceived gradual worsening of their symptoms. As the disease progresses, the decline of the renal function eventually resulted in the outcomes of dialysis. Above feelings will be aggravated gradually [42]. Similarly, our results also showed that the prevalence of depressive symptoms was gradually increased along with the progress of disease. In stages I–V, depression was 8.1%, 24.4%, 20.0%, 37.5%, and 56.3%, respectively. About sixty percent of ESRD symptoms would be complicated by depressive symptoms. Young et al. [4] suggested further testing of targeted depression interventions should be considered in this population, for the reason that major depression at baseline was associated with a 3-fold greater risk of mortality among Stage V CKD diabetic patients.

At the end, there are several limitations in our research that should be addressed. First, our sample was selected from one hospital in China for convenience. The study cannot be considered as representative of the T2DM-related CKD population in China. Second, we used self-reported and closed-ended questionnaires. Third, the number of complications and treatments of ESRD were not considered. Fourth, the sample size in this study is relatively smaller than some other studies.

In conclusion, T2DM-related CKD patients showed a higher predisposition to depression. Medical staff should pay more attention to the psychological issues when T2DM patients were involved in the treatment of CKD, especially for female patients in late stage complicated with hypertension.

## Figures and Tables

**(a):** 

Variables	Overall sample	Depression status		*χ* ^2^/*z*	*P* value
		HAD-D score < 11	HAD-D score ≥ 11		
Total, *n* (%)	210	165 (78.6)	45 (21.4)		
Gender, *n* (%)				7.507	**0.006**
Male	108 (51.4)	93 (86.1)	15 (13.9)		
Female	102 (45.1)	72 (70.6)	30 (29.4)		
Age (year), mean (SD)	57.66 (11.68)	57.39 (11.57)	58.62 (12.14)	−0.624^*∗*^	0.533
Employment status, *n* (%)				1.118	0.290
Working	102 (48.6)	77 (75.5)	25 (24.5)		
Not working	108 (51.4)	88 (81.5)	20 (18.5)		
Education, *n* (%)				3.791	0.150
Primary or below (0–6 years)	65 (31.0)	46 (76.7)	19 (29.2)		
Secondary (7–13 years)	122 (58.1)	99 (79.2)	23 (18.9)		
Tertiary (>13 years)	23 (11.0)	20 (80.0)	3 (13.0)		
Marital status (married), *n* (%)	189 (90.0)	152 (80.4)	37 (19.6)	2.828	0.093
Yearly income (RMB), *n* (%)				8.229	**0.016**
<15000	42 (20.0)	28 (66.7)	14 (33.3)		
15000~33000	75 (35.7)	56 (74.7)	19 (25.3)		
>33000	93 (44.3)	81 (87.1)	12 (12.9)		
Insurance, *n* (%)				2.452	0.117
Rural medicare cooperative	64 (30.5)	46 (71.9)	18 (28.1)		
Medical insurance	146 (69.5)	119 (81.5)	27 (18.5)		

Bold values indicate significant results (*P* < 0.05).

^*∗*^Independent *t*-tests.

**(b):** 

Variables	Overall sample	Depression Status		*χ* ^2^/*z*	*P* value
		HAD-D score < 11	HAD-D score ≥ 11		
clinic visit frequency (times/year), median (IQR)	0.5 (1.0)	0.0 (1.0)	1.00 (2.0)	−2.356	**0.018**
blood glucose testing (times/month), median (IQR)	1.0 (4.0)	1.0 (4.0)	2.0 (3.0)	−2.336	**0.019**
Duration of diabetes (years), median (IQR)	7.0 (12.0)	6.0 (12.92)	8.0 (10.5)	−2.111^*∗*^	**0.035**
Insulin use (yes), *n* (%)	116 (55.2)	94 (81.0)	22 (19.0)	0.934	0.334
Hypertension (yes), *n* (%)	115 (54.8)	83 (72.2)	32 (27.8)	6.180	**0.013**
HbA1c				0.010	0.921
≤9	78 (37.1)	61 (78.2)	17 (21.8)		
>9	132 (62.9)	104 (78.8)	28 (21.2)		
eGFR (mL/min/1.73 m^2^), *n* (%)				15.420	<**0.001**
≥90	75 (35.7)	68 (90.7)	7 (9.3)		
60–89	105 (50.0)	80 (76.2)	25 (23.8)		
<60	30 (14.3)	17 (56.7)	13 (43.3)		
Albuminuria (mg/L), *n* (%)				17.485	<**0.001**
Normal Albuminuria (<20)	109 (51.9)	96 (88.1)	13 (11.9)		
Microalbuminuria (20~200)	59 (28.1)	45 (76.3)	14 (23.7)		
Macroalbuminuria (>200)	42 (20.0)	24 (57.1)	18 (42.9)		
Severity of DKD, *n* (%)				23.283	<**0.001**
Stage I	74 (35.2)	68 (91.9)	6 (8.1)		
Stage II	41 (19.5)	31 (75.6)	10 (24.4)		
Stage III	55 (26.2)	44 (80.0)	11 (20.0)		
Stage IV	24 (11.4)	15 (62.5)	9 (37.5)		
Stage V	16 (7.6)	7 (43.8)	9 (56.3)		

Bold values indicate significant results (*P* < 0.05).

^*∗*^Mann-Whitney test.

**Table 2:** Logistic regression model of T2DM-related CKD patients on depressive symptoms.

Variables	*B*	SE	Wald	OR	exp (*B*) 95% CI		*P* value
					Lower bound	Upper bound	
Hypertension							
Without hypertension	—	—	—	REF	—	—	—
With hypertension	0.902	0.395	5.208	2.465	1.136	5.350	**0.022**
Severity of T2DM-related CKD			16.779				**0.002**
Stage I	—	—	—	REF	—	—	—
Stage II	1.056	0.576	3.357	2.875	0.929	8.899	0.067
Stage III	0.837	0.558	2.252	2.309	0.774	6.886	0.133
Stage IV	1.877	0.622	9.095	6.533	1.929	22.122	**0.003**
Stage V	2.464	0.682	13.056	11.755	3.088	44.744	**0.000**
Gender							
Female	0.993	0.387	6.586	2.699	1.264	5.760	**0.010**
Male	—	—	—	REF	—	—	—

REF: reference group.

Bold values indicate significant results (*P* < 0.05).

**Table 3:** Comparison between the HAD-D score ≥ 11 group and HAD-D score < 11 group in SF-36 domain score.

QOL assessments (SF-36 domains), mean (SD)	Overall sample	HAD-D score < 11	HAD-D score ≥ 11	*Z*	*P* value
PF	85.52 (23.62)	88.86 (19.83)	59.55 (33.45)	−4.685	**<0.001**
RP	62.82 (44.42)	67.40 (42.71)	27.27 (42.19)	−3.649	**<0.001**
BP	85.81 (22.63)	87.30 (22.19)	74.27 (23.25)	−2.929	**0.003**
GH	57.28 (24.33)	60.47 (23.23)	32.50 (17.85)	−4.919	**<0.001**
VT	65.03 (19.01)	66.96 (18.36)	50.00 (17.53)	−3.933	**<0.001**
SF	91.26 (18.76)	94.23 (14.17)	68.18 (31.04)	−5.033	**<0.001**
RE	89.12 (30.66)	93.18 (25.29)	57.58 (47.34)	−4.969	**<0.001**
MH	82.32 (11.72)	84.40 (8.38)	66.18 (19.47)	−4.714	**<0.001**
HT	39.38 (18.33)	40.94 (18.14)	27.27 (15.25)	−3.185	**0.001**
PCS	72.82 (21.70)	75.96 (19.83)	48.40 (20.47)	−5.056	**<0.001**
MCS	81.90 (15.28)	84.66 (11.77)	60.48 (21.71)	−5.994	**<0.001**

Total score	71.54 (13.20)	73.93 (11.00)	53.01 (14.36)	−5.790	**<0.001**

Bold values indicate significant results (*P* < 0.05).

PCS: the physical component summary.

MCS: the mental component summary.
